# The Physical Effects of Aromatherapy in Alleviating Work-Related Stress on Elementary School Teachers in Taiwan

**DOI:** 10.1155/2013/853809

**Published:** 2013-10-21

**Authors:** Shing-Hong Liu, Tzu-Hsin Lin, Kang-Ming Chang

**Affiliations:** ^1^Department of Computer Science and Information Engineering, Chaoyang University of Technology, Taichung, Taiwan; ^2^Department of Cardiology, Lin-Shin Hospital, Taichung, Taiwan; ^3^Department of Photonics and Communication Engineering, Asia University, Taichung, Taiwan; ^4^Graduate Institute of Clinical Medical Science, China Medical University, Taichung, Taiwan

## Abstract

People use aromatherapy to relieve the symptoms of physical and psychological stress. However, previous studies have not precisely clarified a scientific basis for the beneficial effects of aromatherapy. Therefore, the overall purpose of this study was to elucidate the beneficial effect of aromatherapy in relieving work-related stress. Twenty-nine elementary school teachers from Taiwan participated in this study. The experimental procedures comprised 2 phases. First, we verified the effect of aromatherapy by conducting 2 blind tests. We used natural bergamot essential oil extracted from plants and synthesized a chemical essential oil as the placebo to do the aromatherapy. Second, we analyzed the performance of the aromatherapy treatment on the teachers who had various workloads. We measured the teachers' heart rate variability to evaluate their autonomic nervous system activity. The results show that only the natural bergamot essential oil had an effect and that the aromatherapy treatment relieved work-related stress of teachers with various workloads. However, the aromatherapy treatment had a weak effect on young teachers who had a heavy workload. Moreover, the aromatherapy treatment exhibited no effect on teachers who belong to the abnormal body mass index subgroup having a heavy workload.

## 1. Introduction

In modern society, job-related stress is a substantial problem because 40%–50% of all relative work misses are related to stress [1]. Several previous studies have shown that the level of work-related stress could increase the risk of diseases such as cardiovascular disease [2, 3], neurodegenerative diseases [4], chronic diseases of aging [5], and metabolic syndrome [6]. Elementary school teachers in Taiwan must address problems related to Taiwan's low birth rate. In numerous Taiwanese families, there is only one child. Therefore, elementary school teachers must provide more time to process the child's problem except teaching. Moreover, teachers also do various administrative duties. Therefore, all elementary school teachers are at risk of suffering from work-related stress [7]. Thus, it is crucial for elementary schools to identify appropriate methods for coping with stress. 

Various methods for coping with stress have been studied [8]. Aromatherapy is an appealing method because it is both effective and easy to implement [9]. Numerous previous studies have reported using essential oils to alleviate the symptoms of physical and psychological stress [10–13]. Among these studies, bergamot, lavender, and geranium were the most commonly applied essential oils, and the inhalation method was used more than other methods of delivery. These studies employed a randomized controlled trial to conduct the experiments and measured changes in the autonomic nervous systems of participants to quantify the performance of the aromatherapy treatment. During the experimental procedure, the participants were seated for 5 minutes after commencing the test, and their physiological data were collected. Subsequently, the aromatherapy group received the aromatherapy intervention for approximately 10–20 minutes, whereas the control group was either resting or inhaling from an empty diffuser. Following the test, the participants' data were collected. As discussed, the control group did not always use the placebo during the aromatherapy treatment for relieving physical stress symptoms. However, Lorig and Schwartz examined the relationship between the time-domain electroencephalograph (EEG) activity and self-reports from people exposed to various odorants [14]. The results indicate that the odors generated few perceptual or mood differences. EEG alpha and theta are the activity in the left and right hemispheres of the brain. Bagetta et al. used the brain wave spectrum power and found that bergamot essential oil correlates well with its exocytotic and carrier-mediated release of discrete amino acids endowed with the neurotransmitter function in the mammalian hippocampus [15]. Therefore, we considered the placebo a necessary factor for evaluating the effects of aromatherapy treatment.

The autonomic nervous system includes sympathetic and parasympathetic systems. When people feel physical or psychological stress, the sympathetic system becomes more active. When this stressor disappears, the parasympathetic system reduces the heart rate and the breathing rate. The response of the autonomic nervous system can be monitored using the heart rate variability (HRV), which is derived from heartbeat interval time series [16, 17]. Some studies have shown that physical tasks influence the HRV [1, 18, 19]. Psychological depressive and anxiety disorders can also affect the HRV [20, 21]. Therefore, during aromatherapy treatment, the HRV is used to verify the effects of aromatherapy on physical and psychological tasks [10–13, 22]. 

Chang and Shen examined the effect of aromatherapy on elementary school teachers [10]. In their study, two conditions were not considered. (a) A placebo was not used in the experiment. They analyzed the HRV of subjects before and after aromatherapy treatment. If a synthetic essential oil is used in the treatment process, can it alleviate the symptoms of physical and psychological stress? (b) The varying workloads of subjects were not considered when conducting aromatherapy. Thus, quantifying the effect of aromatherapy is difficult.

The overall objective of our study was to clarify the beneficial effects of aromatherapy in alleviating work stress. The experiment comprised two phases. The purpose of the first phase was to verify the effect of aromatherapy by using two blind tests. We used natural essential oil extracted from plants and synthetic essential oil made with chemical materials to do the aromatherapy. The second phase involved analyzing the performance of aromatherapy for participants with varying workloads. To compare our results with those obtained by Chang and Shen, we also recruited elementary school teachers in Taiwan, and natural bergamot essential oil was used during treatment. 

## 2. Materials and Methods

### 2.1. Participants

We recruited 29 elementary schoolteachers who did not have asthma, hypertension, or a heart condition. Because an aromatherapy spray may induce asthma, participants with a history of asthma were excluded. Moreover, numerous studies have shown that heart conditions such as arrhythmia, myocardial ischemia, and a history of heart failure affect the HRV [23–26]. Thus, participants with a heart disease and hypertension also were excluded from this study. The Beck Anxiety Inventory (BAI) was used to estimate the anxiety degree of every participant. The anxiety degree is considered minor when the score ranges between 0 and 7. A score ranging between 8 and 15 indicates a light degree. A score ranging between 16 and 25 indicates a moderate degree, and a score between 26 and 63 indicates a serious degree. 

### 2.2. Outcome Measures

For this study, we used 100% pure bergamot essential oil made in Italy, diluted to 2%. The placebo was a synthetic essential oil (Shunyi Chemical Co., Ltd., Taiwan) with a similar scent to the bergamot essential oil. An ultrasonic ionizer aromatherapy diffuser was used for aroma evaporation (type YHL668/I, ultrasound frequency 2.5 MHz, Nature Creart Co., Ltd, Taiwan). For the heart rate measurements and HRV analysis, we employed a handheld HRV meter (LR8Z11, made by Yunyin Co., Ltd., Taiwan). It measures one lead electrocardiogram (ECG), the sampling rate is 500 Hz, and the resolution is 12 bits. The blood pressure was measured using an electronic blood pressure monitor (type HEM-7210, made by OMRON Co., Ltd., Japan).

The HRV is derived from the heartbeat interval time series whose resampling is 4 Hz by using the further discrete Fourier transform. Low-frequency power (LF: 0.04–0.15 Hz), high-frequency power (HF: 0.15–0.4 Hz), and the logarithmic ratio low- to high-frequency power (LF/HF) were calculated. We also used the normalized LF (LF%) and HF (HF%) to indicate the response of autonomic nervous activity. LF is affected by the vagal nervous and the sympathetic nervous, and HF is affected by the parasympathetic nervous [17].

### 2.3. Experiment Procedure

This experiment was approved by the Asia University Medical Research Ethics Committee. This was a two-phase experiment. The purpose of the first phase was to verify the effect of aromatherapy by conducting a blind test. The second experiment involved analyzing the effect of aromatherapy on teachers with varying workloads. 

#### 2.3.1. The Effect of Aromatherapy

In this experiment, each participant underwent the aroma treatment twice. Although the scent of the natural and synthetic essential oils is similar, certain people who carefully compare them at the same time can distinguish between them. Therefore, we set an interval of one week between the two experiments. To avoid the effect of different workloads, the aroma treatment for each participant was conducted on the same weekday at the same time. In both experiments, the participant did not know which essential oil was used. The experiment procedure is described below.

**Step 1**

Each participant was required to complete a consent form and a BAI survey and provide personal information including gender, age, years of employment, height, weight, and relationship status. Participants were required to abstain from smoking, alcohol, and coffee 6 hours before aroma treatment. At the resting time, the participants were then asked to sit in a chair and rest for approximately 3 minutes.

**Step 2**

Their blood pressure was measured. We then measured the ECG lasting 3 minutes as a pretest. The participants kept their eyes open and remained still.

**Step 3**

The aroma treatment lasted approximately 15 minutes. The respiration rate and volume during this time were asked the same as the resting time.

**Step 4**

Blood pressure was again measured after completion of the aroma treatment. We then measured the ECG lasting 3 minutes as a posttest.

#### 2.3.2. The Performance of Aromatherapy

In this experiment, we recruited the same participants. Each participant underwent aroma treatment twice: once during a heavy-workload state, and once more during a light-workload state. A heavy workload was defined as teaching more than four classes in one day, whereas a light workload was defined as teaching less than two classes in one day. All aroma treatment was conducted after the end of a day's classes. To allow independence for the two treatments, the second treatment was delayed one week from the first treatment. Natural bergamot essential oil was used. The experiment procedure is the same as in Section 2.3.1.

### 2.4. Statistics

We employed the SPSS 12.0 software package to conduct the one-way ANOVA analysis. Significance for the *P* value was set at 0.05. Descriptive statistics were represented as the mean ± standard deviation. LF, HF, LF%, HF%, LF/HF, and the R-R interval time (RRI) were used to indicate the response of the autonomic nervous system. Zhang showed that age and gender affect the HRV [27]. Jarrett et al. indicated that the HRV is affected by the body mass index (BMI) [28]. Therefore, the intergroup differences among age, BMI, and the degree of anxiety were examined using an *F* test. 

## 3. Results

Detailed participant information is shown in Table 1. We examined 3 male participants and 26 female participants. The average age was 41.4 ± 4 years, and the average BMI was 22.2 ± 3.6. The light-anxiety subgroup comprised 9 teachers, and the minor-anxiety subgroup contained 20 teachers. The older subgroup had 7 teachers, and the young subgroup had 18 teachers. Regarding BMI, the abnormal subgroup had 9 teachers, and the normal subgroup had 20 teachers. 

The results obtained after natural bergamot essential oil treatment are shown in Table 2. The LF, LF%, HF%, and LF/HF were found to have a significant difference.  Table 3 shows that the results obtained for the synthetic essential oil did not have any indicators with a significant difference. 

Tables 4 and 5 show the results before and after natural bergamot essential oil treatment for participants with light and heavy workloads, respectively. We can find that LF, LF%, HF%, and LF/HF have significant differences. Therefore, the aromatherapy can alleviate the symptoms of physical and psychological stress at light and heavy workloads. Furthermore, after controlling the different variables, including age, BMI, and anxiety degree, we conducted an analysis of the subgroup regarding the performance of aromatherapy, the results of which are described as follows.  Table 6 shows the results of the age subgroups with a light workload. LF, LF%, HF%, and LF/HF all show a significant difference for the young and older subgroups.  Table 7 shows the results for heavy workload teachers. Only the older subgroup was shown to be affected by aromatherapy, with LF, LF%, HF%, and LF/HF having a significant difference. However, in the young subgroup, none of the indicators, except for LF, were found to have a significant difference.  Table 8 shows the results of the BMI subgroups with a light workload. The indicators, LF, LF%, HF%, and LF/HF, have a significant difference in the normal and abnormal BMI subgroups.  Table 9 shows the results for teachers with a heavy workload. Only the normal BMI subgroup was shown to be affected by aromatherapy; LF, LF%, HF%, and LF/HF have a significant difference in this subgroup. In the abnormal BMI subgroup, none of the indicators showed a significant difference. Tables 10 and 11 show the analyzed results of the anxiety-degree subgroups with light and heavy workloads. LF, LF%, HF%, and LF/HF were all found to have a significant difference.

## 4. Discussions

Chang and Shen examined the effect of aromatherapy on elementary schoolteachers in Taiwan [10]. Although their results were positive, certain research points were not considered clearly, such as placebo use and teachers' varying workloads. The focus of our study was on the physical effect of aromatherapy on elementary schoolteachers with different workloads. We added a placebo variable to determine whether the HRV response was actually obtained from the aromatherapy. The placebo was a synthetic essential oil that has a similar scent to natural bergamot essential oil. We then examined the physical effect of aromatherapy on alleviating the work stress of teachers with different workloads. Because the age, BMI, and anxiety degree could affect the HRV, we also analyzed these subgroups. Chang and Shen used the ANS Watch monitor to measure the HRV and blood pressure. This device measures the pulse wave of the radial artery to detect the HRV. In contrast, we used an ECG to detect the HRV. Thus, the data of certain indicators differ.

In this study, the HF indicator did not show a significant difference in the two experiments. Because the respiration frequency at a resting time is within 0.1~0.4 Hz, which locates at the range of HF, the calculated power of HF will include the power of respiratory frequency [29]. Therefore, some studies of aromatherapy have used only the LH and LF/HF indicators to identify the effect of aromatherapy [10, 13]. In this study, we focused on the LF, LF/HF, LF%, and HF% indicators to analyze the effect and performance of aromatherapy.

According to Tables 2 and 3, although the participants could not distinguish between the scent of the natural essential oil and synthetic essential oil, the natural bergamot essential oil has a relaxing effect, whereas the synthetic essential oil does not. This result directly shows that the essential bergamot oil extracted from natural materials for aromatherapy could be a better choice.

Tables 4 and 5 show the effect of aromatherapy on reducing work stress according to varying workloads. The results are in agreement with those of Chang and Shen [10]. In the age subgroup, the light-workload results are in agreement with those of Chang and Shen [10]. However, the elder subgroup with a heavy workload had a better performance than the young subgroup by the aromatherapy. This result differs from the findings of Chang and Shen because Chang and Shen did not consider differences in subjects' workloads; their results for all age subgroups showed that aromatherapy caused an effect [10]. Zhang demonstrated that age had a greater impact on HRV than gender. The older age group had a consistently lower LF and HF compared with the younger group [27]. In our study, the LF% and LF/HF of the young subgroup decreased, and HF% increased before and after aroma treatment. However, none of these indicators were found to have a significant difference. Therefore, age variable may affect the performance of aromatherapy for teachers with a heavy workload. In the BMI analysis, the aromatherapy had effect on teachers with a light workload. But the HRV did not have the significant change before and after aroma treatment with a heavy workload. This is a very interesting result. Jarrett et al. indicated that age and BMI affect the HRV [28]. However, this was not within the scope of our study.

## 5. Conclusion

We conducted this trial to examine the potential of using natural bergamot essential oil in appeasing the work stress of elementary schoolteachers. The response of automatic nervous system has a significant change after the natural essential bergamot oil treatment. We also analyzed the effect of aromatherapy at different workloads. The aromatherapy may alleviate the symptoms of physical and psychological stress. The results also suggest that age and BMI factors affect aromatherapy performance when teachers have a heavy workload.

## Figures and Tables

**Table 1:** Participant information (*n* = 29).

Items	People number
Gender	Male: 3
	Female: 26
BAI	Minor: 20
	Light: 9
Age (years)	Elder (>40): 18
	Young (≤40): 11
BMI (Kg/m^2^)	>24 : 9
	≤24 and ≥21 : 20

**Table 2:** The HRV one-way ANOVA results before and after natural bergamot essential oil treatment for all participants.

Indicators	Before (*n* = 29)	After (*n* = 29)	*F *	*P *
LF (ms^2^)	9.80 ± 1.19	9.05 ± 1.31	15.21	0.00**
HF (ms^2^)	8.91 ± 1.45	8.96 ± 1.36	0.04	0.840
LF/HF (nu)	0.88 ± 1.00	0.10 ± 1.10	24.34	0.00**
LF% (nu)	68.6 ± 19.4	52.2 ± 22.2	26.76	0.00**
HF% (nu)	32.3 ± 19.5	48.7 ± 22.1	26.92	0.00**
RRI (ms)	766.3 ± 90.2	781.5 ± 98.4	1.12	0.290

*P < 0.05, **P < 0.01.

**Table 3:** The HRV one-way ANOVA results before and after synthetic essential oil treatment for all participants.

Indicators	Before (*n* = 29)	After (*n* = 29)	*F *	*P *
LF (ms^2^)	9.5 ± 1.1	9.3 ± 1.2	0.43	0.511
HF (ms^2^)	8.8 ± 1.2	9.0 ± 1.2	0.99	0.322
LF/HF (nu)	0.64 ± 1.01	0.34 ± 1.13	3.27	0.075
LF% (nu)	63.25 ± 20.11	57.09 ± 22.94	3.54	0.061
HF% (nu)	37.45 ± 20.10	43.64 ± 23.05	3.54	0.061
RRI (ms)	740.1 ± 85.9	760.2 ± 85.7	2.48	0.124

*P < 0.05, **P < 0.01.

**Table 4:** The HRV one-way ANOVA results before and after natural bergamot essential oil treatment for all participants with a light workload.

Indicators	Before (*n* = 29)	After (*n* = 29)	*F *	*P *
LF (ms^2^)	9.74 ± 1.18	8.97 ± 1.30	16.96	0.000**
HF (ms^2^)	8.98 ± 1.49	8.92 ± 1.39	0.07	0.790
LF/HF (nu)	0.76 ± 0.97	0.04 ± 1.14	20.13	0.000**
LF% (nu)	65.85 ± 20.35	50.68 ± 22.57	21.69	0.000**
HF% (nu)	34.45 ± 19.88	50.27 ± 22.43	24.21	0.000**
RRI (ms)	773 ± 96.4	778 ± 102	0.09	0.763

*P < 0.05, **P < 0.01.

**Table 5:** The HRV one-way ANOVA results before and after natural bergamot essential oil treatment for all participants with a heavy workload.

Indicators	Before (*n* = 29)	After (*n* = 29)	*F *	*P *
LF (ms^2^)	9.68 ± 1.15	9.12 ± 1.13	10.41	0.001**
HF (ms^2^)	8.98 ± 1.59	9.26 ± 1.27	1.68	0.196
LF/HF (nu)	0.70 ± 1.15	−0.14 ± 1.16	23.04	0.000**
LF% (nu)	63.6 ± 22.3	47.3 ± 24.0	21.47	0.000**
HF% (nu)	37.2 ± 22.4	53.4 ± 23.9	21.42	0.000**
RRI (ms)	771 ± 114	7821 ± 109	0.36	0.548

*P < 0.05, **P < 0.01.

**Table 6:** The HRV one-way ANOVA results of the age subgroups with a light workload before and after natural bergamot essential oil treatment for all participants.

Indicators	Elder (*n* = 18)				Young (*n* = 11)			
	Before	After	*F *	*P *	Before	After	*F *	*P *
LF (ms^2^)	9.74 ± 1.15	9.06 ± 1.41	7.50	0.007**	9.75 ± 1.25	8.81 ± 1.10	10.42	0.002**
HF (ms^2^)	8.70 ± 1.42	8.70 ± 1.38	0.00	0.995	9.45 ± 1.52	9.29 ± 1.36	0.19	0.660
LF/HF (nu)	1.04 ± 0.82	0.36 ± 1.13	12.71	0.001**	0.30 ± 1.02	−0.48 ± 0.94	10.41	0.002**
LF% (nu)	71.2 ± 17.5	57.1 ± 21.8	13.59	0.000**	57.2 ± 21.9	40.2 ± 19.9	10.92	0.002**
HF% (nu)	28.7 ± 16.5	43.1 ± 21.8	16.61	0.000**	43.8 ± 21.6	60.7 ± 19.6	11.04	0.001**
RRI (ms)	763 ± 74.7	771 ± 76.5	0.36	0.552	792 ± 123	790 ± 136	0.01	0.946

*P < 0.05, **P < 0.01.

**Table 7:** The HRV one-way ANOVA results of the age subgroups with a heavy workload before and after natural bergamot essential oil treatment for all participants.

Indicators	Elder (*n* = 18)				Young (*n* = 11)			
	Before	After	*F *	*P *	Before	After	*F *	*P *
LF (ms^2^)	9.55 ± 1.23	9.08 ± 1.16	4.258	0.042*	9.89 ± 0.98	9.19 ± 1.10	7.32	0.009**
HF (ms^2^)	8.55 ± 1.67	9.11 ± 1.30	3.71	0.057	9.67 ± 1.17	9.51 ± 1.20	0.31	0.583
LF/HF (nu)	1.00 ± 1.18	−0.03 ± 1.07	22.53	0.000**	0. 21 ± 0.91	−0.32 ± 1.30	3.71	0.058
LF% (nu)	69.0 ± 22.2	49.6 ± 22.9	20.02	0.000**	54.8 ± 19.8	43.6 ± 25.7	3.91	0.052
HF% (nu)	31.7 ± 22.4	51.2 ± 22.9	20.15	0.000**	46.1 ± 19.7	57.0 ± 25.4	3.78	0.056
RRI (ms)	759 ± 86.2	769 ± 76.4	0.46	0.498	792 ± 148	801 ± 147	0.07	0.797

*P < 0.05, **P < 0.01.

**Table 8:** The HRV one-way ANOVA results of the BMI subgroups with a light workload before and after natural bergamot essential oil treatment for all participants.

Indicators	BMI > 40 (*n* = 9)				21 ≤ BMI ≤ 24 (*n* = 20)			
	Before	After	*F *	*P *	Before	After	*F *	*P *
LF (ms^2^)	9.7 ± 1.6	8.8 ± 1.6	4.59	0.037*	9.8 ± 0.9	9.1 ± 1.2	13.46	0.000**
HF (ms^2^)	8.71 ± 2.09	8.63 ± 2.05	0.02	0.895	9.11 ± 1.13	9.1 ± 1.0	0.07	0.790
LF/HF (nu)	0.97 ± 0.77	0.12 ± 0.97	12.89	0.001**	0.66 ± 1.04	0.01 ± 1.21	10.16	0.002**
LF% (nu)	71.1 ± 15.4	52.3 ± 20.1	14.95	0.000**	63.5 ± 21.94	50.0 ± 23.7	10.53	0.002**
HF% (nu)	29.9 ± 15.5	48.7 ± 20.4	14.47	0.000**	36.5 ± 21.36	51.0 ± 23.4	12.53	0.001**
RRI (ms)	767 ± 139	773 ± 163	0.02	0.888	777 ± 70.1	781 ± 60.2	0.11	0.740

*P < 0.05, **P < 0.01.

**Table 9:** The HRV one-way ANOVA results of the BMI subgroups with a heavy workload before and after natural bergamot essential oil treatment for all participants.

Indicators	BMI > 40 (*n* = 9)				21 ≤ BMI ≤ 24 (*n* = 20)			
	Before	After	*F *	*P *	Before	After	*F *	*P *
LF (ms^2^)	9.40 ± 1.30	9.33 ± 1.13	0.05	0.817	9.80 ± 1.06	9.02 ± 1.13	14.93	0.000**
HF (ms^2^)	8.88 ± 1.93	8.96 ± 1.48	0.08	0.774	9.04 ± 1.43	9.40 ± 1.16	2.18	0.143
LF/HF (nu)	0.58 ± 0.93	0.37 ± 0.91	0.71	0.402	0.76 ± 1.24	−0.37 ± 1.20	25.60	0.000**
LF% (nu)	62.4 ± 19.1	58. 6 ± 20.1	0.50	0.481	64.2 ± 23.7	42.3 ± 24.1	25.20	0.000**
HF% (nu)	38.7 ± 19.6	42.4 ± 20.3	0.45	0.507	36.4 ± 23.7	58.4 ± 23.8	25.54	0.000**
RRI (ms)	768 ± 153	802 ± 152	0.64	0.426	773 ± 93.0	772 ± 82.9	0.00	0.989

*P < 0.05, **P < 0.01.

**Table 10:** The HRV one-way ANOVA results of the anxiety-degree subgroups with a light workload before and after natural bergamot essential oil treatment for all participants.

Indicators	Minor (*n* = 20)				Light (*n* = 9)			
	Before	After	*F *	*P *	Before	After	*F *	*P *
LF (ms^2^)	9.72 ± 1.24	9.12 ± 1.36	6.27	0.014*	9.80 ± 1.08	8.62 ± 1.10	15.85	0.000**
HF (ms^2^)	8.95 ± 1.54	8.89 ± 1.43	0.04	0.834	9.06 ± 1.40	9.00 ± 1.33	0.03	0.870
LF/HF (nu)	0.77 ± 1.03	0.23 ± 1.24	6.69	0.011*	0.74 ± 0.82	−0.38 ± 0.72	28.01	0.000**
LF% (nu)	65.6 ± 21.7	54.4 ± 24.4	6.94	0.010*	66.5 ± 17.4	42.3 ± 15.0	29.99	0.000**
HF% (nu)	34.3 ± 21.0	46.6 ± 24.3	8.73	0.004**	34.8 ± 17.4	58.5 ± 14.8	29.02	0.000**
RRI (ms)	778 ± 102	788 ± 115	0.24	0.626	763 ± 82.5	756 ± 64.5	0.12	0.733

*P < 0.05, **P < 0.01.

**Table 11:** The HRV one-way ANOVA results of the anxiety-degree subgroups with a heavy workload before and after natural bergamot essential oil treatment for all participants.

Indicators	Minor (*n* = 20)				Light (*n* = 9)			
	Before	After	*F *	*P *	Before	After	*F *	*P *
LF (ms^2^)	9.62 ± 1.27	9.14 ± 1.23	4.28	0.041*	9.82 ± 0.81	9.07 ± 0.88	10.46	0.002**
HF (ms^2^)	8.83 ± 1.66	9.21 ± 1.33	1.91	0.170	9.31 ± 1.40	9.38 ± 1.15	0.04	0.841
LF/HF (nu)	0.79 ± 1.18	−0.06 ± 1.25	14.82	0.000**	0.51 ± 1.08	−0.31 ± 0.95	8.74	0.005**
LF% (nu)	65.3 ± 22.9	49.2 ± 25.3	13.32	0.000**	60.0 ± 20.8	43.2 ± 20.8	8.75	0.005**
HF% (nu)	35.4 ± 23.0	51.6 ± 25.2	13.59	0.000**	41.1 ± 21.1	57.5 ± 20.7	8.30	0.006**
RRI (ms)	77.3 ± 9.5	74.7 ± 11.6	0.08	0.783	68.3 ± 9.6	66.9 ± 9.6	0.94	0.337

*P < 0.05, **P < 0.01.
